# Non-thermal plasma treated solution with potential as a novel therapeutic agent for nasal mucosa regeneration

**DOI:** 10.1038/s41598-018-32077-y

**Published:** 2018-09-13

**Authors:** Ho-Ryun Won, Sung Un Kang, Haeng Jun Kim, Jeon Yeob Jang, Yoo Seob Shin, Chul-Ho Kim

**Affiliations:** 1 Department of Otolaryngology-Head and Neck Surgery, Chungnam National University Hospital, Daejeon, Republic of Korea; 2 Department of Otolaryngology, Ajou University School of Medicine, Suwon, Republic of Korea; 3 Department of Molecular Science and Technology, Ajou University, Suwon, Republic of Korea

## Abstract

Adequate and rapid mucosal regeneration is one of the most important factors in the healing process of nasal mucosa after surgery or trauma. In particular, delayed mucosal regeneration after surgery is an important cause of surgical failure. However, no effective treatment is available yet. Non-thermal plasma (NTP) has several medical effects, but the existing probe type is limited to local direct treatment. Therefore, we investigated the various effects using liquid type plasma to overcome this limitation. In addition, the therapeutic effects of non-thermal plasma treated solution (NTS) on nasal mucosa have yet to be determined. Experiments were carried out using BEAS-2B, a human bronchial epithelial cell line similar to nasal mucosa epithelium. NTS had no cytotoxicity to the BEAS-2B cells and enhanced cell proliferation. NTS also promoted migration of BEAS-2B cells. NTS increased cell proliferation and migration via epidermal growth factor receptor (EGFR) activities and epithelial-to-mesenchymal transition (EMT) signaling. Furthermore, NTS enhanced wound healing of nasal mucosa in an animal model. Accordingly, NTS promotes nasal mucosa wound healing by increasing cell proliferation and migration. These findings suggest the therapeutic potential of NTS in nasal mucosa wound healing.

## Introduction

Nasal disease and associated symptoms are common worldwide, and reportedly occur in about 4.5% to 15% in North America and Europe^1^. Treatment of nasal diseases refractory to medical treatment can be improved by nasal surgery. For example, endoscopic sinus surgery (ESS) is indicated for various nasal diseases such as recurrent acute sinusitis, chronic sinusitis, obstructive nasal polyposis, fungal sinusitis, and peri-orbital abscess^2^. ESS is the most commonly performed procedure, accounting for more than 50% of all ear, nose, and throat operations^3^. Despite advances in nasal surgery, the damage to normal nasal mucosa and the delay in mucosal wound healing after nasal surgery remain significant problems^4,5^. The sino-nasal cavity is composed of ciliated pseudostratified columnar epithelium. It acts as a physical defense against exogenous agents and maintains normal ventilation through a mechanical clearing system. Thus, any damage to the nasal mucosa by the nasal surgery and delayed healing disrupts the physiological function.

Plasma is known as the fourth state of matter. It is a partially ionized gas containing electrons, ions, radicals and energetic photons produced by applying energy to the gas^6^. Plasma medicine, including the medical use of plasma, is a fast-growing field that is potentially novel therapeutic modality. It was recently demonstrated that plasma has anticancer, sterilizing, blood-coagulation, tissue regenerating and anti-inflammatory effects^7–9^. In our previous study, we demonstrated several medical effects of non-thermal plasma (NTP), but the existing probe types has a limit to local direct treatment or epidermis permeability^8,9^. Therefore, a new type of plasma device is needed. We are investigating the effects of liquid type of non-thermal plasma treated solution (NTS). In addition, the therapeutic effects of NTS on nasal mucosa have yet to be determined.

In this study, we evaluated the effect of nasal irrigation with NTS on the nasal mucosa after injury. We also evaluated the mechanism of therapeutic effect of NTS on the nasal mucosal epithelial cells.

## Results

### NTS has no cytotoxicity to the BEAS-2B cells and enhances cell proliferation

**Figure 1:**
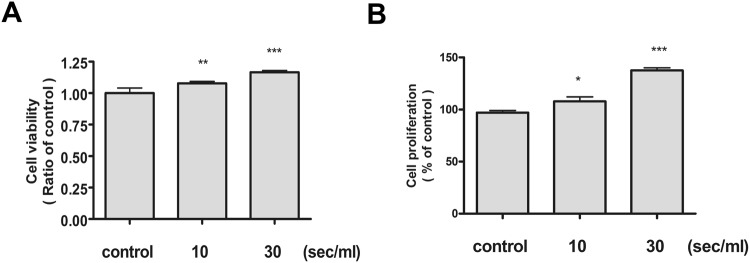
NTS does not induce cytotoxicity in the bronchial epithelial cells and proliferation is increased. (**A**) NTS did not affect bronchial epithelial cells viability, but rather increased in proportion to the treatment time. NTS was generated by NTP treatment for 10 and 30 seconds in culture media, and was measured after culturing for 24 hours. Cell viability was evaluated by the MTT assay. (**B**) NTS increased bronchial epithelial cell proliferation. Statistically significant proliferation was increased with treatment time. Cell proliferation was measured with a BrdU assay. Asterisks indicate statistically significant differences (*P < 0.05; **P < 0.01; ***P < 0.001).

Experiments were conducted using BEAS-2B, a human bronchial epithelial cell line similar to nasal mucosa epithelium. First, the cytotoxicity against NTS was assessed using the MTT assay. No significant cytotoxicity was observed in the NTS group generated by treatment with NTP for 10 and 30 seconds (Fig. 1A). The effect of cell proliferation was also confirmed by the BrdU assay and significant cell proliferation occurred in the NTS-treated group. The degree of proliferation increased in proportion to the NTS treatment time (Fig. 1B). The BrdU assay was performed again using 0.9% NaCl prepared by diluting with the culture medium similar to saline used *in vivo*. Cell proliferation was statistically significant (Sup. Fig. 1A). In other words, it was confirmed that NTS used liquid with different compositions showed the same effect.

### NTS enhances migration of BEAS-2B cells

**Figure 2:**
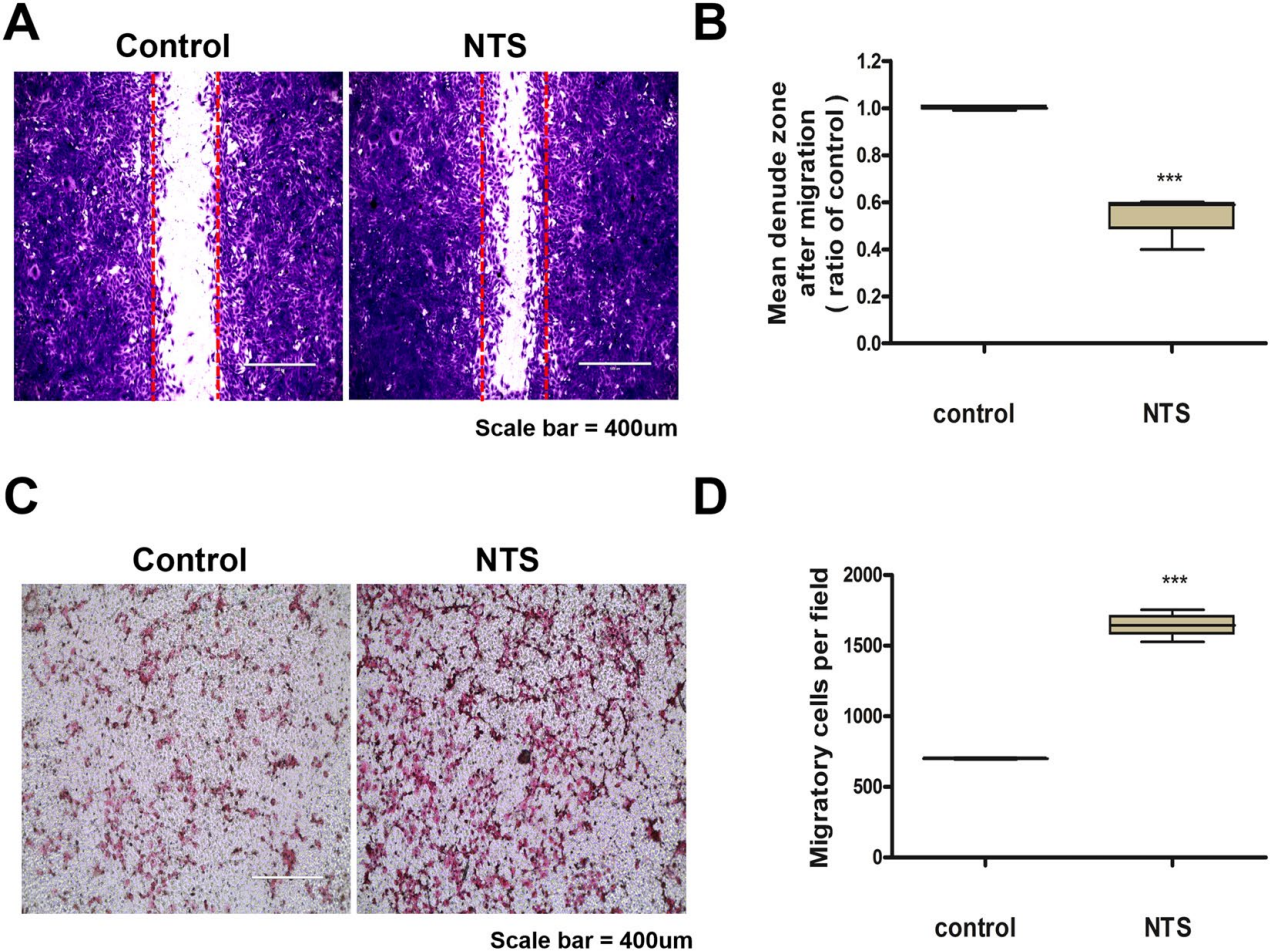
NTS increases cell migration. (**A**) NTS increases cell migration. The cells were treated with NTS generated by NTP for 30 seconds and incubated for 24 hours. BEAS-2B cells were plated in 6-well plates and a scratch wound healing assay was performed. (**B**) NTS decreased the denuded zone of scratched area significantly. Mean denuded zone was obtained by calculating the ratio of average area of the denuded zone to the area in the control. Asterisks indicate statistically significant differences (***P < 0.001). (**C**) Transwell migration assay was performed after NTS treatment under the same conditions. (**D**) The number of migrated cells increased statistically. Cells were photographed using a fluorescence microscope and examined at ×10 magnification. Asterisks indicate statistically significant differences (***P < 0.001).

The degree of cell migration affects the wound healing ability. Therefore, the effect of NTS on cell migration was analyzed with scratch wound healing assay. The increased migration of BEAS-2B cells in the NTS group was confirmed by microscopy (Fig. 2A), and it was confirmed again by the ratio of denuded zone in the scratch area (Fig. 2B). Transwell migration assay was also performed to identify the effect of NTS on cell migration and confirmed that the migration of cells was increased after NTS treatment (Fig. 2C). We quantified and verified the significant increase in migration (Fig. 2D).

### NTS increases cell proliferation and migration via epidermal growth factor receptor (EGFR) activities

**Figure 3:**
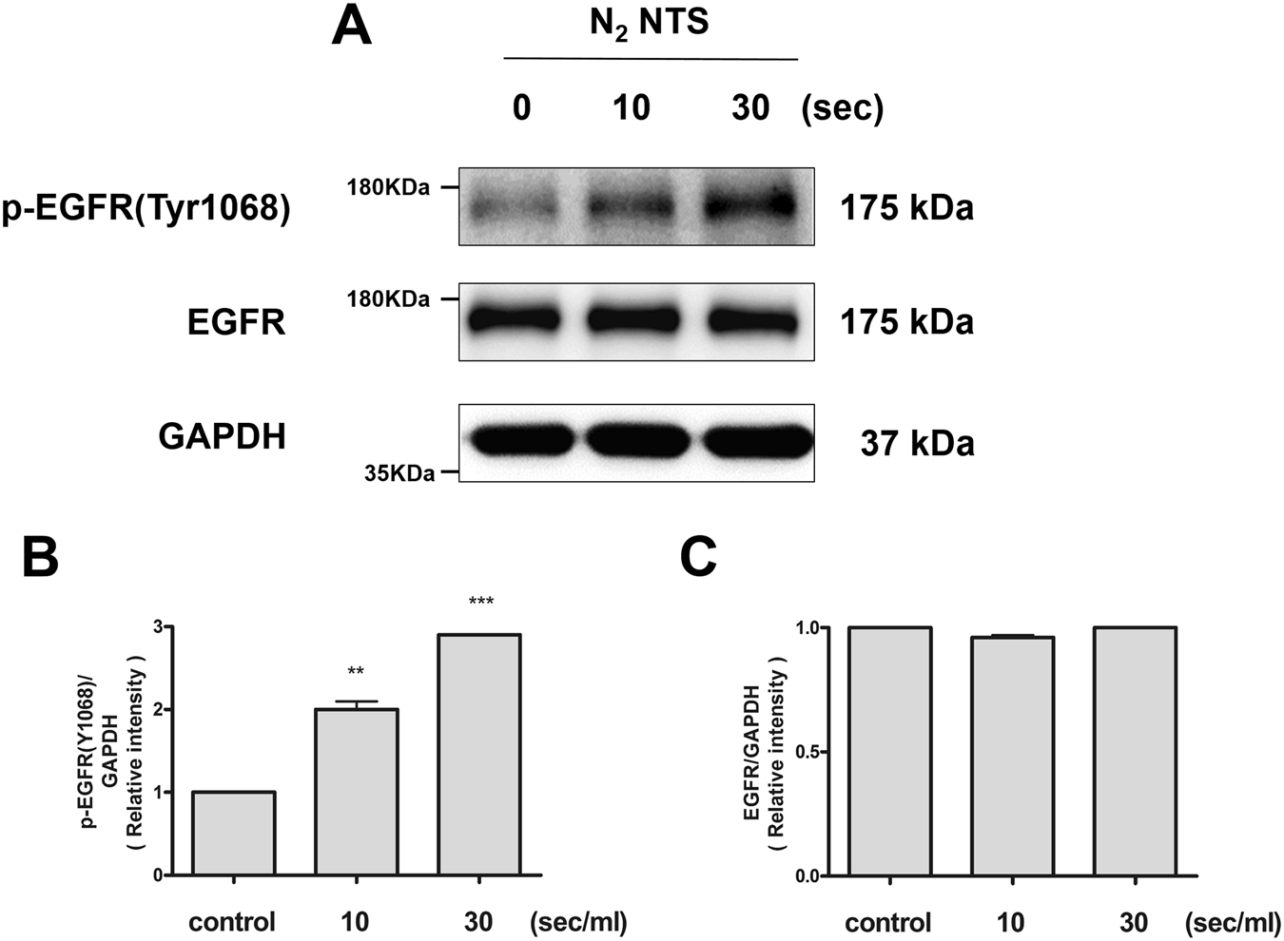
NTS increases EGFR activity, not amounts of EGFR protein. (**A**) NTS increases the expression of p-EGFR. The expression level of p-EGFR and total EGFR was evaluated using the Western blot. NTS was generated by NTP treatment for 10 and 30 seconds. After NTS treatment, the expression level of p-EGFR was increased with NTP treatment time and the total EGFR expression was not changed. The grouping of blots cropped from different parts of the same gel. (**B**) NTS increases the relative intensity of p-EGFR and statistically increased with the treatment time. Asterisks indicate statistically significant differences (**P < 0.01; ***P < 0.001). (**C**) NTS does not affect changes in total EGFR. The total amount of EGFR protein after NTS treatment did not change statistically.

Epidermal growth factor (EGF) is a key factor in the healing of nasal mucosa. EGF promotes differentiation, development and angiogenesis of the nasal mucosa, thereby promoting wound healing^10^. After NTS treatment, the amounts of phospho-EGFR (p-EGFR) and total EGFR proteins were measured by Western blot. The amount of p-EGFR was increased in proportion to the NTP treatment time (Fig. 3A,B). On the other hand, the total EGFR was not affected by NTS (Fig. 3A,C). In other words, NTS affects only the activity of EGFR, suggesting that it promotes cell proliferation and migration.

### NTS increases cell migration via the epithelial-to-mesenchymal transition (EMT) signaling

**Figure 4:**
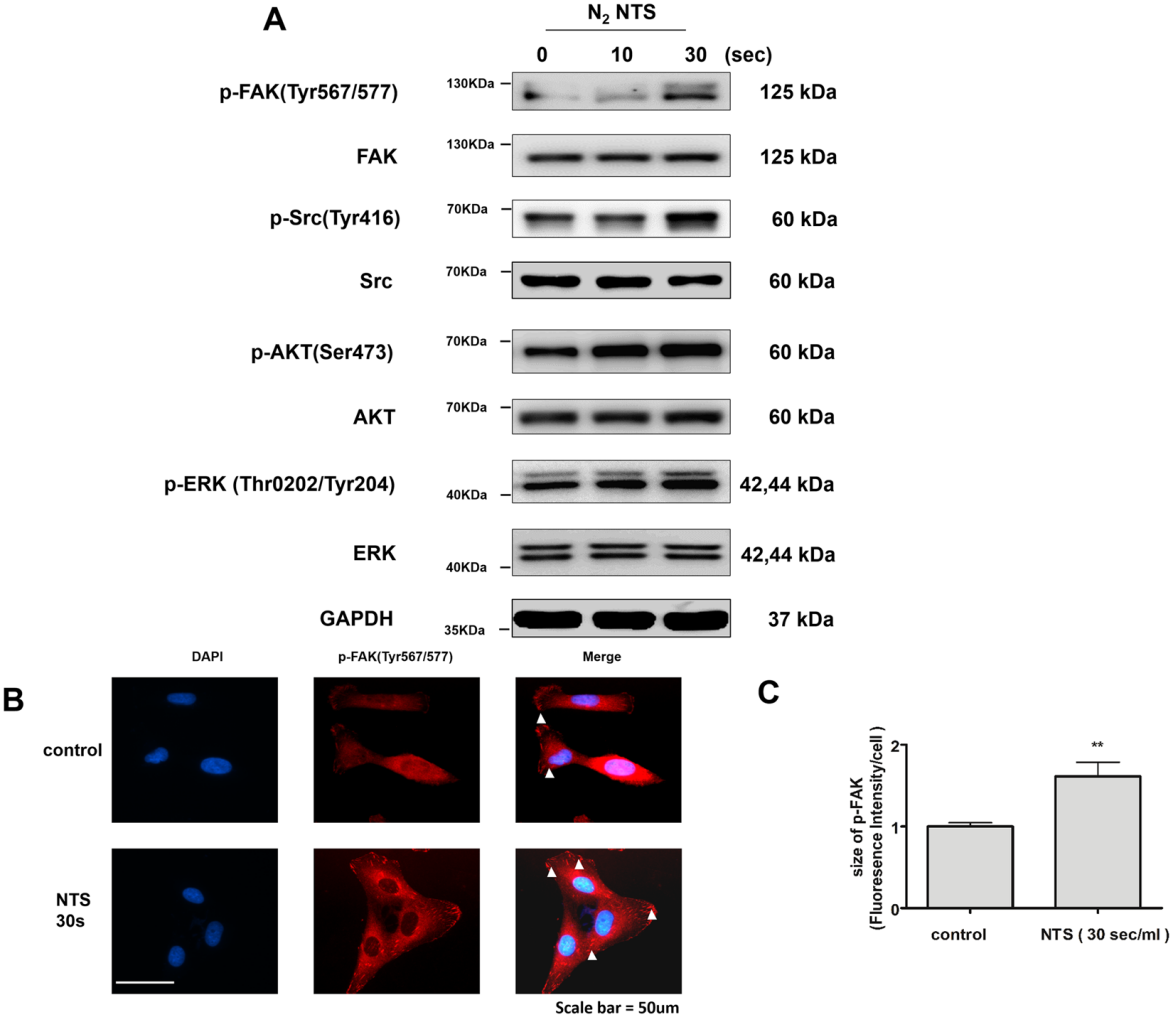
NTS increases the expression of proteins involved in EMT signaling. (**A**) Expression of proteins involved in EMT signaling was confirmed by Western blot. NTS was generated by NTP treatment for 10 and 30 seconds. The degree of protein expression of p-FAK and p-Src was significantly increased with NTP treatment time. The grouping of blots cropped from different parts of the same gel. (**B**) The increase in the expression of p-FAK after NTS treatment was confirmed using immunefluorescence assay. The red-stained stock in cytoplasm represents p-FAK (white arrow). The amount of red- stained stock was increased in the NTS treated group. (**C**) Fluorescence intensity of p-FAK statistically significantly increased by NTS. Asterisks indicate statistically significant differences (**P < 0.01).

**Figure 5:**
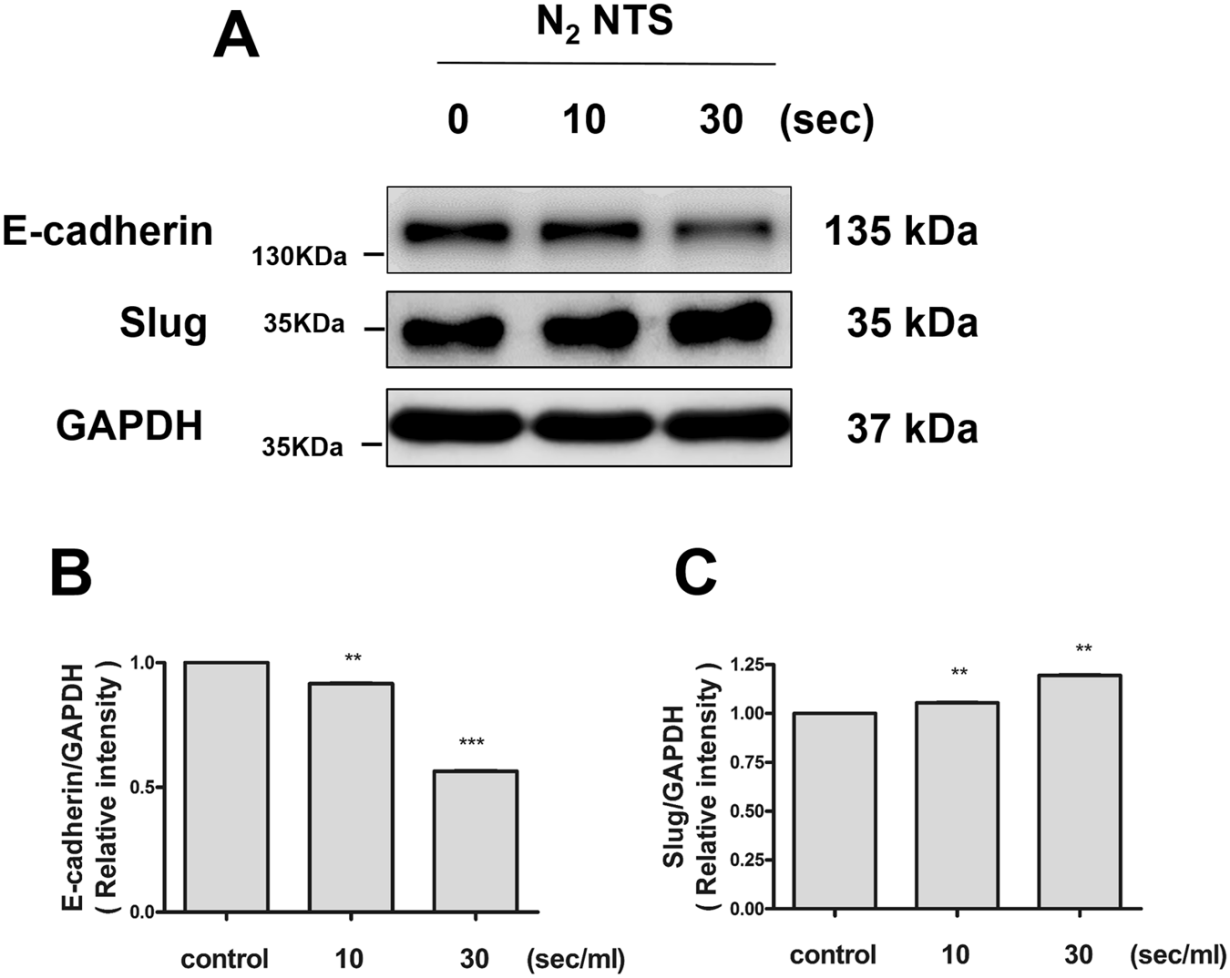
NTS reduces the expression of E-cadherin, while the expression of slug increases. (**A**) NTS induces down-regulation of E-cadherin and up-regulation of Slug. NTS was generated by NTP treatment for 10 and 30 seconds. The degree of expression of E-cadherin and slug was confirmed by Western blot. The grouping of blots cropped from different parts of the same gel. (**B,C**) Relative intensity was identified and confirmed again. Relative intensity of E-cadherin decreased with NTP treatment time, while slug increased. Both values were statistically significant. Asterisks indicate statistically significant differences (**P < 0.01; ***P < 0.001).

Activation of the EMT signaling pathway during the cutaneous wound healing induces cell migration^11^. The expression of proteins regulating EMT was confirmed by Western blot. In the NTS group generated by NTP treatment for 30 seconds, the level of phosphor-Src (p-Src) and phospho-FAK (p-FAK) were found to increase significantly. The expression of phospho-AKT (p-AKT) and phospho-ERK (p-ERK) also increased slightly (Fig. 4A). The increase of p-FAK was confirmed again by immune-fluorescence assay (Fig. 4B). The result was quantified using fluorescence intensity and statistically significant increase was confirmed. (Fig. 4C). E-cadherin is a major molecule that mediating cell-to-cell adhesion, and thus plays an important role in cell migration. Therefore, the down-regulation of E-cadherin is a hallmark of the EMT^12^. The down-regulation of E-cadherin after NTS treatment was observed by Western blot (Fig. 5A,B). Slug, a well-known mesenchymal marker, was also found to increase after NTS treatment (Fig. 5A,C). EMT-related proteins were also found to be similar result to NTS generated with 0.9% NaCl diluted in the culture medium (Sup. Fig. 1B–D). In conclusion, NTS promotes cell migration by increasing EMT signaling.

### NTS enhances matrix metalloproteinases-2 (MMP-2)/matrix metalloproteinases-9 (MMP-9) activities

**Figure 6:**
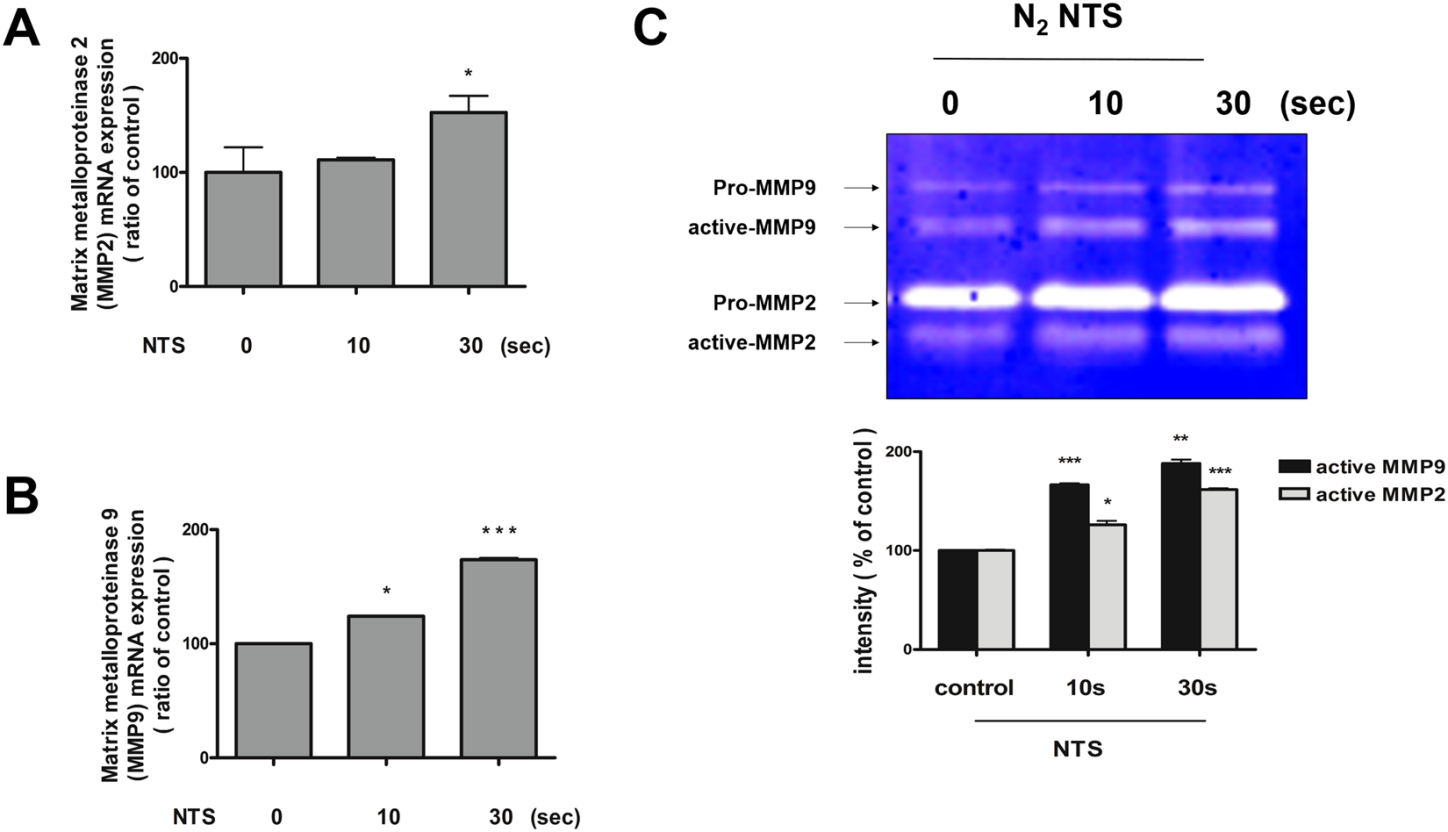
NTS activates MMP-2/MMP-9. (**A,B**) NTS increases mRNA expression of MMP-2/MMP-9. NTS was generated by NTP treatment for 10 and 30 seconds. MMP-2/MMP-9 mRNA level was measured using real-time PCR. The mRNA expression of MMP-2/MMP-9 was significantly increased in the group treated with NTP for 30 seconds. Asterisks indicate statistically significant differences (*P < 0.05; ***P < 0.001). (**C**) NTS increases MMP-2/MMP-9 activity in bronchial epithelial cells. Gelatin zymography for MMP-2/MMP-9. Only MMP-2/MMP-9 of active form was statistically increased after NTS treatment. Asterisks indicate statistically significant differences (*P < 0.05; **P < 0.01; ***P < 0.001).

MMP-2/MMP-9 also plays an important role in the wound healing process, which induces cell migration. Therefore, the activity of MMP-2/MMP-9 was measured after NTS treatment using a zymogram and real-time PCR. MMP-2/MMP-9 mRNA expression was significantly augmented after NTS treatment compared with that of the control (Fig. 6A,B) Moreover, MMP-2/MMP-9 activities also increased after NTS treatment (Fig. 6C), suggesting that NTS induces migratory and remodeling events during wound healing by increasing not only MMP-2/MMP-9 transcription but also the enzymatic activity of existing MMP-2/MMP-9.

### NTS enhances nasal mucosa wound healing in an animal model

**Figure 7:**
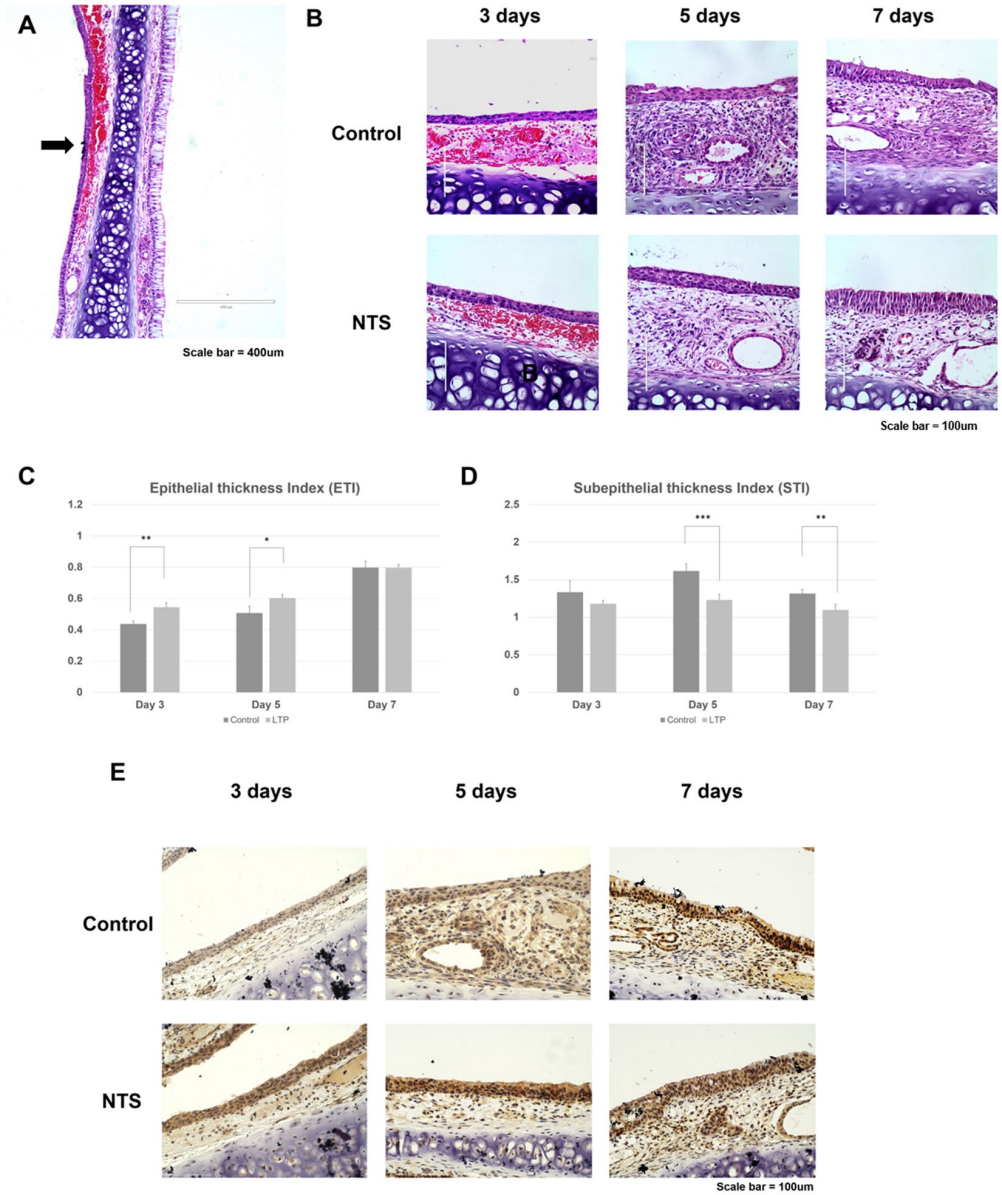
NTS improves the regeneration of nasal mucosal epithelium in animal models of nasal wound. (**A**) H&E staining of septal mucosa after nasal wound formation using nasal brushing technique. The basement membrane was well maintained and the wound was properly formed (black arrow). (**B**) H&E staining of septal mucosa after normal saline (control) and NTS treatment. NTS was generated by NTP treatment for 30 seconds. The thickness of the epithelial layer was increased in the NTS group. However, the thickness of the subepithelial layer was reduced. (**C**) ETI increased post-NTS treatment. Compared with the control group, the NTS treated group showed a statistically significant increase in ETI. (**D**) After NTS treatment, STI increased. Starting with day 5 of NTS treatment, STI was significantly decreased in NTS treated group. Asterisks indicate statistically significant differences (*P < 0.05; **P < 0.01; ***P < 0.001). (**E**) Immuno-histochemical staining of septal mucosa after normal saline and NTS treatment. Higher expression of p-EGFR was observed in the NTS group.

First, the right nasal septal mucosa was wounded with a nasal brushing technique and confirmed by microscopy. The basement membrane was retained and the wound was induced on the epithelium of the right septal mucosa (Fig. 7A). The rats were sacrificed on days 3, 5, and 7 after NTS treatment, and both sides of the septal mucosa were observed at high magnification. On day 3, the epithelial thickness was increased more in the NTS group, and the difference from the control group increased significantly with the passage of time (Fig. 7B). Starting with day 5, the number of nuclei in the epithelial cells proliferating from the basement membrane was increased in the NTS treated group, which increased further during the 7 days after treatment (Fig. 7B). In addition, the thickness of lamina propria, a sub-epithelial layer, was increased from day 5 after wound formation, reflecting edematous changes. Sub-epithelial thickness was slightly decreased in the NTS treated group, which also showed similar findings on the 7 days after treatment. Epithelial thickness index (ETI) and sub-epithelial thickness index (STI) were obtained by calculating the ratio of average height of the newly formation to the height in the normal side. ETI was significantly increased up to 5 days after NTS treatment, suggesting increased epithelial proliferation compared with the control group (Fig. 7C). Furthermore, STI was significantly decreased in the NTS treated group from day 5 until day 7 after NTS treatment (Fig. 7D). This is suggesting a decrease in the edematous change of the NTS treated group compared with the control group. In addition, inflammatory cell infiltration of the dermis during the whole observation period showed a tendency to decrease overall in the NTS treated group compared with the control group. The expression of p-EGFR in the tissues was confirmed by immune-histochemical analysis. During the treatment period, it was confirmed that the expression of p-EGFR was high in the NTS group (Fig. 7E).

## Discussion

Nasal surgery is an important procedure in the treatment of nasal diseases. Especially, ESS has been the most effective treatment for patients with pathological problems in the sino-nasal structure during the past 2 decades^13^. Therefore, it was performed in patients with sino-nasal disease refractory to medication. Recent study reported that 64% of patients undergoing ESS showed improved quality of life over the long term^14^. Although nasal surgery is an effective treatment modality, the delayed healing of nasal mucosa and the formation of synechiae induce potential treatment failure in nasal surgery. Particularly, about 20% of patients have problems associated with delayed nasal mucosal healing after nasal surgery, which may require additional surgery^4,15^.

Many studies have been performed to improve the recovery of nasal mucosa after surgery. Several studies have showed that systemic steroid administration before and after surgery was effective against nasal polyps and also promoted healing of nasal mucosa^16^. However, long-term systemic administration of steroids may have a negative effect on the hypothalamic-pituitary-adrenal axis function and a few studies have shown negative impact on mucosal ciliary regeneration^17^. We previously demonstrated that NTP enhances wound healing and muscle regeneration^8,9^. We also implemented NTS to overcome the limitations of local treatment using NTP device. In this study, we evaluated the effect and the mechanism of NTS on the nasal mucosa, and confirmed the possibility of improved healing of the nasal mucosa delayed after surgery.

Postoperative wound healing mechanism of the nasal mucosal lining is complex, highly organized and well-coordinated and is mediated via inflammation, cell proliferation, matrix deposition, and tissue remodeling^17,18^. During the recovery process, the extracellular matrix of nasal mucosa directly affects various growth factors, thereby increasing their receptors and controlling cell phenotype and adhesion^19^. Of the various growth factors, epidermal growth factor family is representative, and includes EGF, amphiregulin, and transforming growth factor-α. EGF involved wound healing process by inducing chemotaxis, mitosis, and epithelial differentiation targeting epithelial cells^10^. In this experiment, the activity of EGFR in the upper airway epithelial cell was increased after NTS treatment. As a result, proliferation and migration were increased.

*In vitro* models of nasal wound healing have reported that epithelial cell migration and spread during the first 12 hours are important for wound healing^20^. This finding was also confirmed in animal models, and the migration persisted up to about 1 week after wound formation^18^. EMT was originally described as an important mechanism for the formation of mesenchymal cells in mesoderm from primitive epithelium during the process of gastrulation^21^. It is involved in renal, pulmonary, cardiac and liver fibrosis, cutaneous scleroderma^22^, and cancer metastasis^23^. EMT in cutaneous wound healing is still a controversial issue^11^. However, the loss of physiological adhesion of epithelial cells by EMT and the increased mobility are essential for migration^22^.

Our previous study demonstrated that NTP treatment improves cutaneous wound healing by modulating EMT to promote migration^9^. Therefore, this study focus EMT modulation in nasal wound healing. Among the proteins involved in regulation of EMT, p-Src and p-FAK were significantly increased in NTS treated bronchial epithelial cells and the mesenchymal marker Slug was also increased. In addition, E-cadherin, a typical cell adhesion marker, was also decreased. Cell adhesion plays an important role in migration in the wound healing process and is regulated by special molecules located on the cell surface^12,24^. During the migration of the epithelial cells, cellular detachment occurs due to the regulation of these molecules^25^. Typically, the down-regulation of E-cadherin occurs^26^. Conversion of motile cells due to the loss of these adherent epithelial phenotypes is also an important factor in the EMT process^22^. Consequently, migration of NTS treated bronchial epithelial cells was increased, and was mediated by E-cadherin downregulation and EMT signaling.

MMPs are proteinases that play a pivotal role in cell migration and tissue remodeling by modifying the wound matrix^27^. After wound formation, ECM components such as type 4 collagen, fibronectin, or elastin, and cellular debris around the wound are metabolized by MMPs for degradation in the matrix associated with wound healing^28,29^. In particular, MMP-2/MMP-9 are gelatinases, which play an important role in cell migration during wound healing^27^. In this experiment, the expression of MMP-2/MMP-9 was increased after NTS treatment resulting in the promotion of cell migration.

We used human bronchial epithelial cells to determine the effect of NTS on nasal mucosa. The bronchial mucosa is composed of pseudostratified epithelium, a characteristic form of the upper airway mucosa^30^. It also has cilia, similar in morphology and function to the nasal mucosa. Although the bronchial epithelial cells are similar to the epithelium of the upper airway tract with the nasal mucosa, it has a differential origin, which may a limitation of this study. Future experiments using primary cultured cells of the human nasal mucosa are necessary.

It has been reported in the literature that the process of nasal mucosa wound healing in rats proceeds in the following order^31^. First, there is an increase in neutrophils at 2 days after wound formation followed by reepithelization on day 5. Reepithelization is manifested by undifferentiated epithelial cells or squamous epithelium, with a decrease in neutrophils and an increase in the number of monocytes. In addition, fibroblasts accumulate in the lamina propria composed of collagen matrix. Goblet cells and ciliated cells begin to form on day 14 after wound formation and regeneration of the normal mucosa occurs on day 28. In particular, cell migration has been reported to occur actively from the beginning of wound formation and lasts up to 168 hours^18^. *In vitro* experiments showed that the effects of proliferation and migration were enhanced. To confirm the effect of proliferation and migration, we carried out *in vivo* experiments for 7 days and confirmed significant promoting of nasal wound healing. In addition, a decrease in edema of lamina propria and a down-regulation in aggregation of inflammatory cells were observed. However, we did not observe the complete regeneration time of the mucous membrane. In addition, we could not confirm the regeneration effect of the goblet or ciliated cells, which is a limitation of this study. Therefore, long term *in vivo* experiments and additional *in vitro* experiments are needed to elucidate the mechanisms and effects in future experiments. Furthermore, NTS is expected to have several effects on the inflammatory reaction of wound in the nasal mucosa, and additionally on the inflammatory disease occurring in the nasal mucosa, warranting additional investigation.

It has been reported that the character is different depending on the composition of the liquid used to make the NTS by treating NTP with the liquid^32^. *In vitro*, we performed NTP treatment on culture media for cell survival. However, in clinical, normal saline (0.9% NaCl) is used for nasal irrigation. Therefore, NTP treatment was used for saline *in vivo* experiments. Therefore, additional experiments were performed to confirm that the characteristics of NTS were changed. H_2_O_2_, pH and NO (Nitric oxide) were measured for NTP-treated NaCl solution and NTP-treated medium solution. In the case of NaCl solution, both the saline used in the *in vivo* study and the 0.9% NaCl prepared by diluting with the culture medium were measured and compared with NTP-treated medium. In the case of pH and H_2_O_2_, the baseline values of the control group in saline differed in culture media. Thus, different results were obtained after the NTP treatment. However, in the diluted NaCl solution, the results were similar to NTP-treated medium. NO was significantly increased after plasma treatment in both saline, 0.9% NaCl diluted with the culture medium and culture media (Sup. Fig. 2). During H_2_O_2_ inhibitor NAC (N-acetylcysteine), increased cell migration and cell proliferation by NTS were not decreased. However, cell migration and cell proliferation were decreased by NO inhibitor PITO (2-Phenyl-4,4,5,5-tetramethylimidazoline-1-oxyl 3-oxide) treatment (Sup. Fig. 2). In this study, it was confirmed that NO affecting the effect of NTS increases regardless of the liquid used in the experiment. However, NO cannot explain all of the NTS effect. It is unclear what constitutes the medical effects of NTP reported in various literature, and this is the major limitation of NTP.

In conclusion, NTS promotes wound healing via epithelial cell proliferation and migration of nasal mucosa suggesting potential therapeutic agents to promote nasal mucosal healing through inhalation or irrigation based on the intervention at the time of injury such as surgery or after trauma. It is also expected to serve as a treatment for pathological morphology of upper airway mucosa such as nasal mucosa.

## Methods

### Cell culture

BEAS-2B human bronchial epithelial cells were purchased from the American Type culture collection (ATCC, Manassas, VA, USA). The cells were maintained in Dulbecco’s Modified Eagle’s Medium (DMEM, GIBCO, Carlsbad, CA, USA) supplemented with 10% Fetal Bovine Serum and 100 U/ml penicillin-streptomycin at (Gibco, Paisley, PA, USA) at 37 °C with 5% CO_2_ under humidified conditions.

### Design of non-thermal plasma treated solution (NTS)

In this study, the NTP system was designed based on our previous study on its biological research applications^8,33^. We used nitrogen (N_2_) as carrier gases for the plasma treated solution because we found that it improved cell permeation efficiency inside the medium^34–36^. For NTS treatment, we added 10 ml of solution to a petri-dish (100 mm diameter, TPP, Renner, Dannstadt, Germany). The distance between the plasma device and the bottom of the petri dish was about 4 cm. NTS treatment time was 10, and 30 seconds per ml. Specifications of the power supply with this system were: 7~8 kV, and mean frequency 25 kHz. For this study, 3~4 kV power was used.

### Cell cytotoxicity activity (MTT assay)

To evaluate whether NTS treatment affects cellular cytotoxicity, MTT (3-(4,5-dimethylthiazol-2-yl)-2,5-diphenyl-tetrazolium bromide, Sigma-Aldrich, St Louis, MO, USA) was used as described previously^8^. Briefly, BEAS-2B cells were seeded in 96-well cell culture plate at a density of 2 × 10^3^ cells/well. After 24 hours, cells were treated with NTS. Cell viability results were presented as ratio to untreated cells.

### Cell proliferation activity (BrdU assay)

To measure the cell proliferation activity of NTS against BEAS-2B cells, 4 × 10^3^ cells/well were seeded on 96-well plates, incubated for 24 hours. NTP was treated on the solution for 10 and 30 seconds and cultured for another 24 hours. Cell proliferation was measured with a BrdU assay kit (Roche Diagnostics, Penzberg, Germany). Cells were incubated with a reagent following the manufacturer’s instructions. Absorbance was measured at wavelength of 370 nm using an ELISA reader (Bio–Tek, Winooski, VT, USA). The rate of cell proliferation was expressed as a percentage of the untreated cells.

### Scratch wound healing assay

Cells were plated in 6-well culture plates at a density of approximately 1 × 10^5^/well and grown to confluence. Scratch wound healing assays were performed as described previously^9^. Briefly, the monolayer was scratched with a sterile pipette tip, followed by extensive washing to remove cellular debris. The cells were treated with NTS, after 24 hours incubation at 37 °C, and photographed using a light microscope (EVOS FL Auto cell imaging system, Thermo Fisher Scientific, Waltham, MA, USA) in five random fields.

### Transwell migration assay

For transwell migration assay, using 24-Transwell chamber (Costar, Cambridge, MA, USA) with a polystyrene membrane (8 μm pore size). Initially, fibronectin (2 mg/filter) was dissolved in 100 ml of DMEM and poured into the upper part of the polyethylene filter (pore size, 8 mm). The wells were coated for 1hrs and incubated at 37 °C with 5% CO_2_. Then, 2 × 10^5^ cells (in 50 μl of growth medium) were seeded on the top of the filter in the upper chamber with or without NTS testing. The lower compartment contained 600 μL of growth media supplemented with fibronectin. The chamber was incubated for 24 hours in 5% CO_2_ at 37 °C. Finally, the attached cells in the lower section were stained with H&E, and counted using light microscopy.

### Western blot

All the Western blotting experiments were performed under the same condition. Cells were lysed in RIPA buffer (Sigma Aldrich, St. Louis, MO, USA) containing 150 mM NaCl, 1.0% Nonidet-P 40, 0.5% sodium deoxycholate, 0.1% sodium dodecyl sulfate, 50 mM Tri (pH 8.0), complete EDTA-free protease inhibitor, and PhoSTOP (Roche Molecular Biochemicals, Basel, Switzerland) as described previously^9^. After cell lysates were prepared, run on a 10∼12% SDS-PAGE gel. After transferring the blots onto PVDF membranes, we immediately cropped the targeted blots according to referenced indicating markers, and then targeted proteins were immunoblotted with its specific antibody for normalization of protein. The following primary antibodies were used: phospho-FAK(y567/577), FAK, phospho-Src(y416), Src, Phospho-AKT(ser473), AKT, phospho-ERK(Thr202/Tyr204), ERK, phospho-EGFR(Y1068), EGFR, and GAPDH(1:1000; Cell Signaling Technology, Danvers, MA, USA). Secondary antibodies (anti-rabbit IgG or anti-mouse IgG, 1:2000) were purchased from Cell Signaling Technology (Danvers, MA, USA). Immunoreactivity of specific proteins was detected using ECL Western blotting kit (GE, Hercules, CA, USA) according to the manufacturer’s instructions.

### Immunofluorescence assay

BEAS-2B cells were cultured on coverslips (Thermo Fisher Scientific, Rochester, NY, USA), differentiated, and treated with NTS (30 seconds/ml) or vehicle control. At 24 hours after incubation, cells were fixed with 4% formaldehyde and blocked with 5% BSA (bovine serum albumin, Millipore, Bedford, MA, USA) in phosphate-buffered saline (PBS) for 1 hour. Cover-slipped and incubated with polyclonal rabbit anti-p-FAK (1:200; Cell Signaling Technology, Danvers, MA, USA) for 2 hours, washed with PBS, and incubated with Alexa 488-labeded antibody (1:500, Molecular Probe, Eugene, Oregon, CA, USA) for 1 hour. After washing three times with PBS, the slides were stained with Hoechst 33258 (Molecular Probe) and incubated at room temperature for 5 minutes to counterstain the nuclei. Slides were washed with PBS, mounted with Vectashield (Vector laboratories, Inc., Burlingame, CA, USA), and visualized using a fluorescence microscope (EVOS FL Auto, Thermo Fisher Scientific, Waltham, MA, USA).

### Quantitative real-time PCR

Total RNA was extracted from BEAS-2B cells treated with NTS using TRIzol®reagent (Gibco-BRL, Grand Island, NY, USA). Total RNA (1 μg) was mixed with 10 μl of ReverTrace qPCR RT (Toyobo Co. Ltd., Osaka, Japan) mixture for cDNA synthesis according to the manufacturer’s instruction. Targeted genes were quantified with one-step real-time PCR using StepOnePlus^TM^(Applied Biosystems, Foster City, CA). The following specific primers were used: MMP-2 Forward, 5′-TTCAGCTCTGGGATGACCTT-3′; MMP-2 Reverse, 5′-CAAGGTGCTGGCTGAGTAGATC-3′, MMP-9 Forward, 5′-TTGACAGCGACAAGAAGTGG-3′; MMP-9 Reverse 5′-GCCATTCACGTCGTCCTTAT-3′, GAPDH Forward 5′-AGGGCTGCTTTTAACTCTGGT-3′; GAPDH Reverse 5′-CCCCACTTGATTTTGGAGGGA-3′.

### Gelatin zymogram assay

Zymogram activities were assayed using gelatin zymography, as described previously^9^. Cells were treated with NTS only for 10 to 30 seconds and incubated for an additional 24 hours. The supernatant (100 μl) from each sample was mixed with 1 ml of 100 mM 4-aminophenylmercuric acetate (Sigma-Aldrich), and the samples were incubated for 1 hour at 37 °C. The sample was placed in sample buffer (without Mecatoeti) for 10 minutes and electrophoresed on a polyacrylamide gel at 125 V for 120 minutes at 4 °C using a Novex Xcell II system (Life Technologies, Carlsbad, CA, USA). The gels were incubated in renaturation buffer for 60 minutes at room temperature, followed by incubation for 18 hours in 100 ml of developing buffer at 37 °C under gentle shaking. The gels were then stained for 3 hours with Coomassie Brilliant Blue. After decolourization in 400 ml of methanol, 100 ml of acetic acid, and 500 ml of distilled water, images were obtained using an image analyzer.

### *In vivo* study

Eighteen healthy male Sprague–Dawley rats, 8 to 10 weeks of age and weighing 200–250 g were acclimatized for 1 week at 21°C  ±  1°C, 50% ± 5% humidity, and an automatic 12-hours light/dark cycle. The rats were provided access to food and water freely. Animal care and procedures were in accordance with the National Institutes of Health

Guidelines for the Care and Use of Laboratory Animals, and all experiments were approved by the Committee for Ethics in Animal Experiments of the Ajou University School of Medicine.

To avoid irritation of the nasal mucosa, anaesthesia was performed with i.m. injection of Zoletile 50 (10 mg/kg; Vibac, Carros, France). Mechanical injuries were performed with an interdental brush (10 mm) inserted through the right nostril^31^.

Eighteen rats were allocated randomly to two groups of nine rats each: (1) a saline (0.9% NaCl) group, and (2) a NTS group. NTS was generated by NTP treatment for 30 seconds per ml in normal saline. In each group, 0.1 ml of saline and 0.1 ml of NTS were instilled in the right nasal cavity for 7 days once a day. Instillation was performed under anaesthesia, and it was deposited as a bead of fluid on the external nares and the rats were allowed to aspirate it. After instillation, the breath was carefully monitored to prevent respiratory failure^37^. Three rats from each group were sacrificed on days 3, 5, and 7 after the mechanical injury (Sup. Fig. 4). The maxilla (including the sinonasal cavity) was extracted for tissue processing, and the specimens were fixed in 4% neutral-buffered formalin solution and decalcified in Decalcifying Solution-Lite (Sigma-Aldrich, Saint-Louis, USA) overnight. The specimens were excised from the posterior part of the upper incisors to the incisive papilla^38^ and processed according to standard paraffin-embedding procedures. After preparing the tissue slides, 5 slides from each specimen were selected randomly and the histopathological changes in the nasal mucosa were determined by H&E. Images were acquired and analyzed using a light microscope. The acquired images were analyzed using Image J software (National Institutes of Health, Maryland, USA).

### Immuno-histochemical analysis

Immuno-histochemistry was performed with paraffin-embedded nasal mucosa tissue sections. The paraffin-embedded samples were sectioned serially into 6 μm thick segments. Briefly, the sections were stained with anti-EGFR antibody (1:500; Cell Signaling Technology, Danvers, MA, USA) overnight at 4 °C. The sections were rinsed in PBS and incubated for 2 hours at room temperature with secondary antibody. Images were analyzed using a light microscope (Nikon E600; Nikon, Tokyo, Japan).

### Statistical analysis

We conducted one-way analysis of variance (ANOVA) based on Mann-Whitney U test using SPSS 20.0 statistical software (SPSS, Chicago, IL, USA). In the *in vitro* experiment, parameters of the data from three independent experiments were expressed as the mean ± S.D. Data from *in vivo* experiments were expressed as mean ± SEM. *P* < 0.05 was considered to statistically significant (*P < 0.05; **P < 0.01; ***P < 0.001).

### Ethical statement

This study was conducted under the approval of the Institutional Animal Experiment Committee at Ajou University School of Medicine (IACUC number: 2016-0031).

## Electronic supplementary material

Supplementary figure

Supplementary Dataset 1

Supplementary Dataset 2

Supplementary Dataset 3

Supplementary Dataset 4

Supplementary Dataset 5
