# Supplementary material for: Non-thermal plasma treated solution with potential as a novel therapeutic agent for nasal mucosa regeneration
Source: Sci Rep. 2018 Sep 13;8:13754. doi: 10.1038/s41598-018-32077-y (PMC6137218; doi:10.1038/s41598-018-32077-y)
Supplement: Supplementary file 1 — Supplementary figure [file 41598_2018_32077_MOESM1_ESM.pdf]

Non-thermal plasma treated solution with potential as a novel therapeutic agent for nasal mucosa regeneration

Ho-Ryun Won, MD[1†], Sung Un Kang, PhD[2†], Haeng Jun Kim, MS[2], Jeon Yeob Jang, MD, PhD[2], Yoo Seob Shin, MD, PhD[2,3], Chul-Ho Kim, MD, PhD[2,3*]

Supplement Figure 1

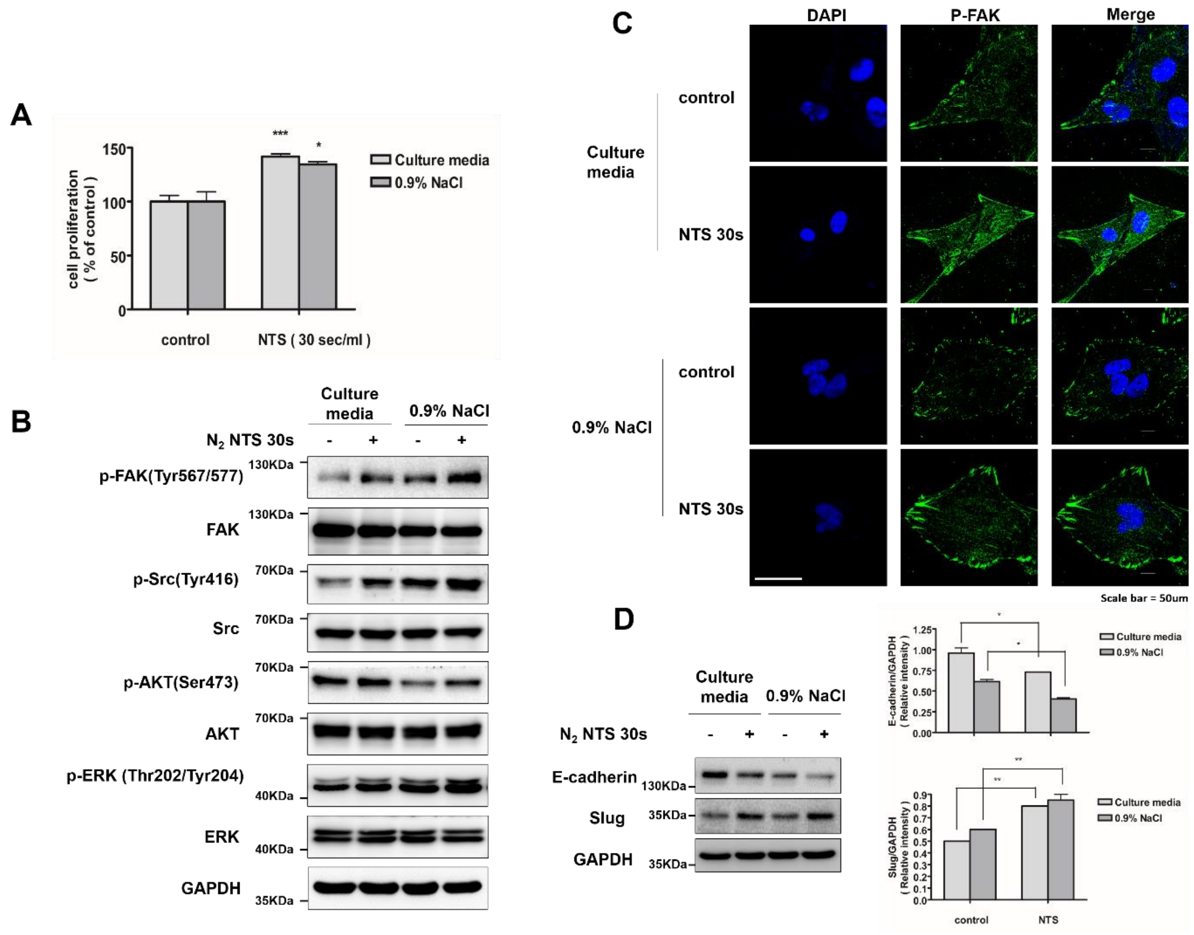

Sup. Fig. 1. Cell proliferation and EMT-related protein effects of NTS generated by 0.9% NaCl diluted with the culture media. (a) NTS increased bronchial epithelial cell proliferation. NTS was generated by NTP treatment for 30 seconds in 0.9% NaCl diluted with the culture media. Cell proliferation was measured with a BrdU assay. Asterisks indicate statistically significant differences (*P < 0.05; ***P < 0.001). (b) The degree of expression of EMT- related proteins was significantly increased with NTP treatment time. NTS was generated by NTP treatment for 30 seconds. (c) The increase in the expression of p-FAK after NTS treatment was confirmed using immunefluorescence assay. Similar result was obtained regardless of the type of NTS. (d) NTS induces down-regulation of E-cadherin and up-regulation of Slug. NTS was generated by NTP treatment for 30 seconds in 0.9% NaCl diluted with the culture media. Both values were statistically significant. Asterisks indicate statistically significant differences (*P < 0.05; **P < 0.01).

Supplement Figure 2

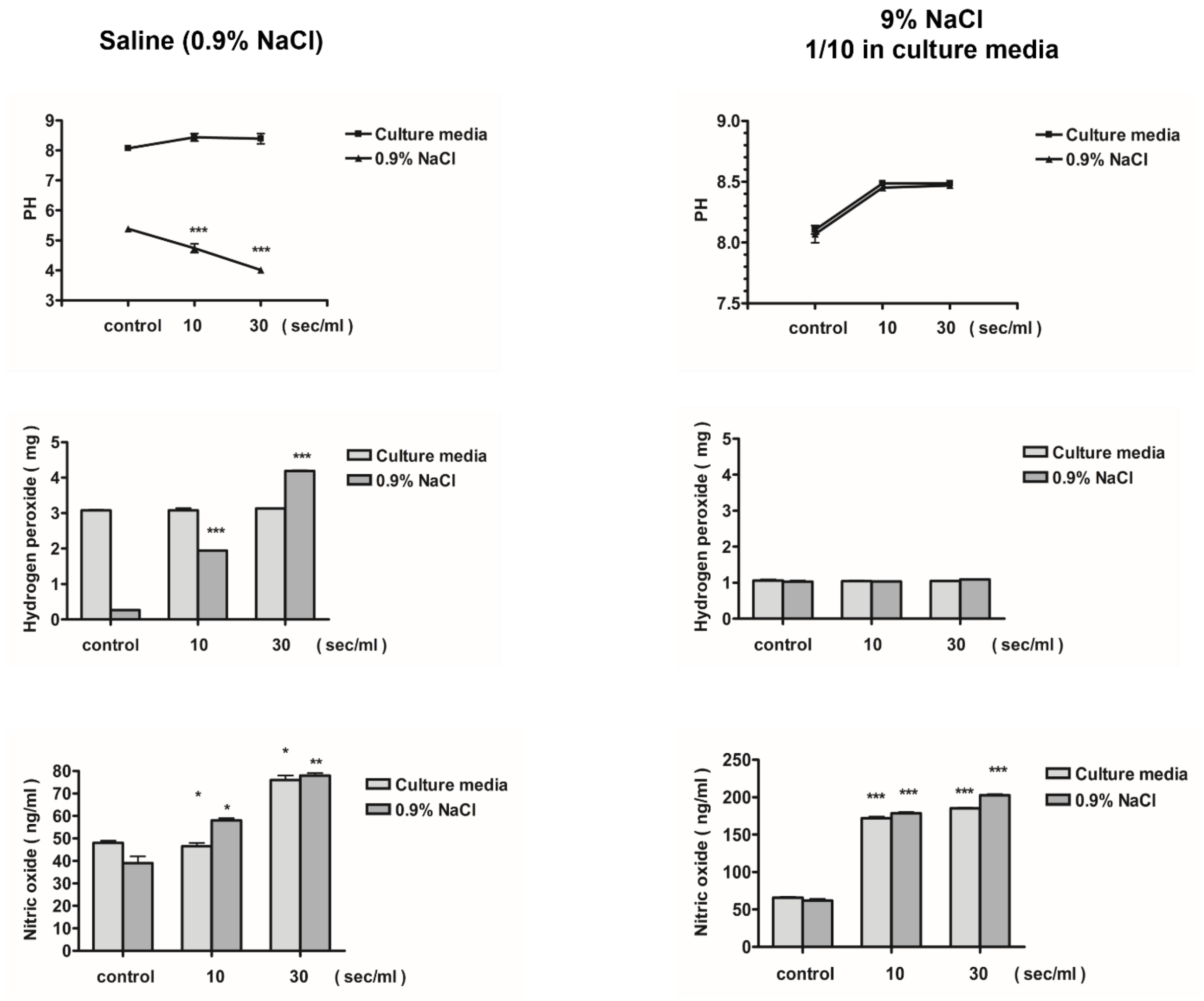

Sup. Fig. 2. The character is different depending on the composition of the liquid used to make the NTS by treating NTP with the liquid. Both the saline used in the in vivo study and the 0.9% NaCl prepared by diluting with the culture medium were measured and compared with NTP-treated medium. The values of the pH and H_2O_2 were different after NTP treatment between saline and culture media. In the diluted NaCl solution, the results were similar to NTP-treated medium. NO was significantly increased after plasma treatment in both saline, 0.9% NaCl diluted with the culture medium and culture media. Asterisks indicate statistically significant differences (*$P < 0.05$; **$P < 0.01$; ***$P < 0.001$).

Supplement Figure 3

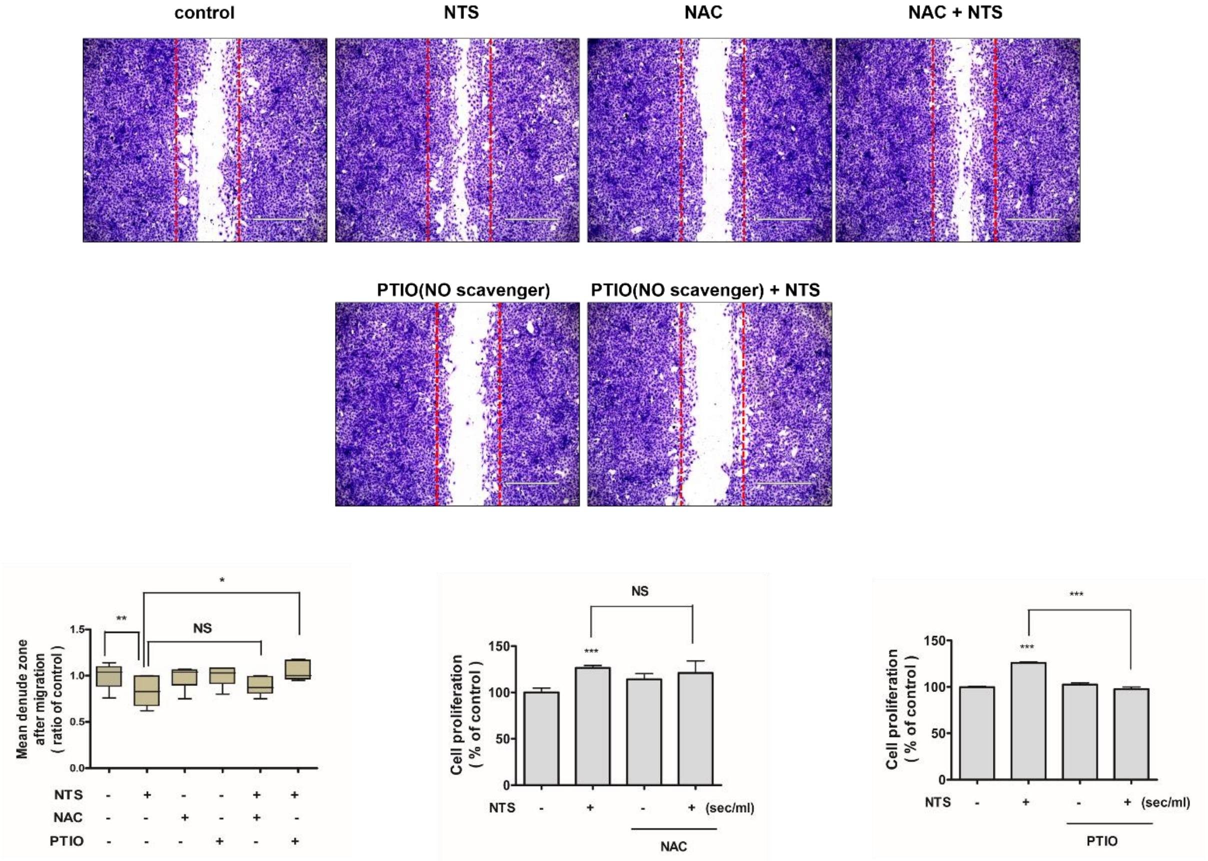

Sup. Fig. 3. The effect of NTS is due to NO produced by NTS. Experiments were carried out using H2O2 scavenger NAC and NO scavenger PTIO. During H2O2 inhibitor NAC, increased cell migration and cell proliferation by NTS were not decreased. Cell migration and cell proliferation were decreased by NO inhibitor PITO treatment. Scratch wound healing assay and BrdU were performed. NTS was generated by NTP treatment for 30 seconds in culture medium. Asterisks indicate statistically significant differences (*P < 0.05; **P < 0.01; ***P < 0.001).

Supplement Figure 4

Control group

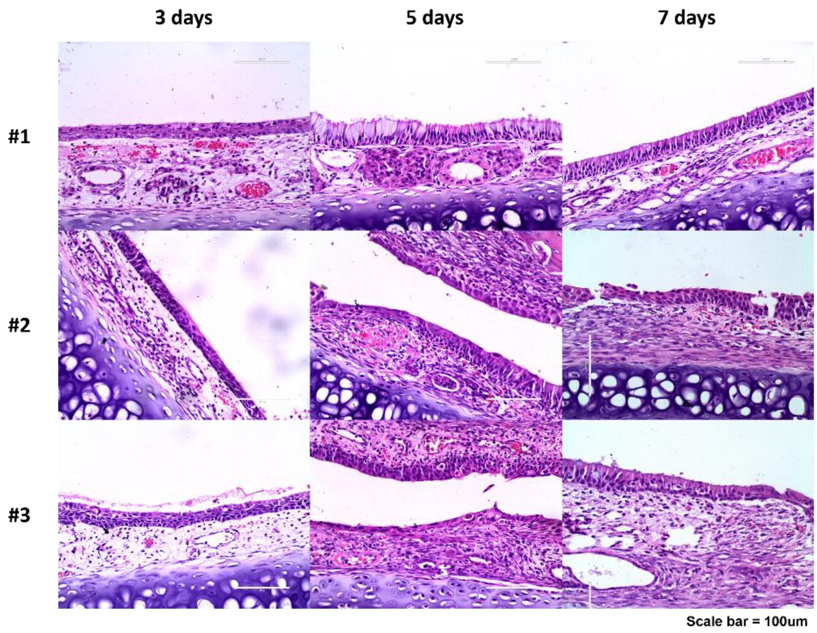

NTS group

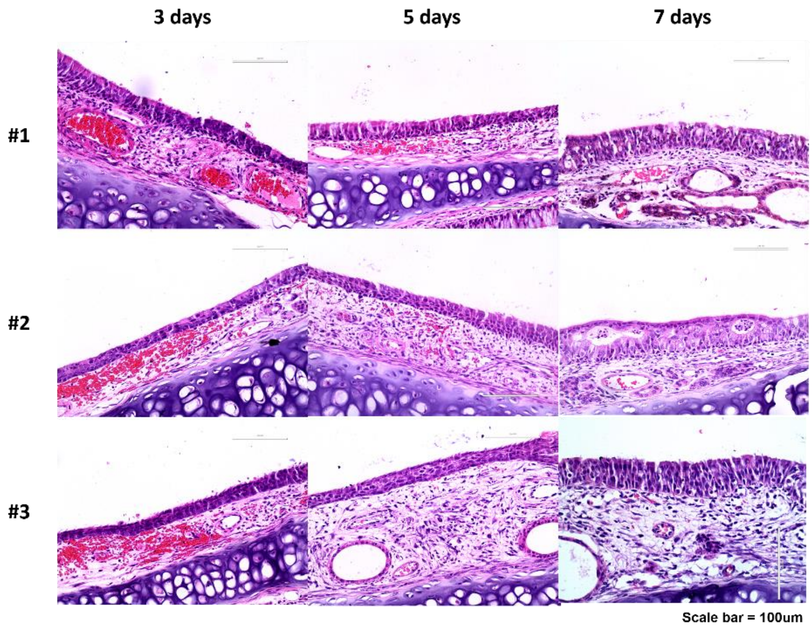

Sup. Fig. 4. H&E sections of all animals per group after normal saline (control) and NTS treatment.
